# Pan-Genome Analyses of the Genus *Cohnella* and Proposal of the Novel Species *Cohnella silvisoli* sp. nov., Isolated from Forest Soil

**DOI:** 10.3390/microorganisms11112726

**Published:** 2023-11-08

**Authors:** Chunling Wang, Lutian Mao, Gegen Bao, Honghui Zhu

**Affiliations:** 1 College of Life Science, Huizhou University, Huizhou 516007, China; wangcl@hzu.edu.cn (C.W.); mlt@hzu.edu.cn (L.M.); 2 Guangzhou Key Laboratory for Research and Development of Crop Germplasm Resources, Zhongkai University of Agriculture and Engineering, Guangzhou 510225, China; baogegen@126.com; 3 Key Laboratory of Agricultural Microbiomics and Precision Application (MARA), Provincial Key Laboratory of Microbial Culture Collection and Application, Key Laboratory of Agricultural Microbiome (MARA), State Key Laboratory of Applied Microbiology Southern China, Institute of Microbiology, Guangdong Academy of Sciences, Guangzhou 510642, China

**Keywords:** isolation, *Cohnella silvisoli*, taxonomic studies, pan-genome

## Abstract

Two strains, designated NL03-T5^T^ and NL03-T5-1, were isolated from a soil sample collected from the Nanling National Forests, Guangdong Province, PR China. The two strains were Gram-stain-positive, aerobic, rod-shaped and had lophotrichous flagellation. Strain NL03-T5^T^ could secrete extracellular mucus whereas NL03-T5-1 could not. Phylogenetic analysis based on 16S rRNA gene sequences revealed that the two strains belong to the genus *Cohnella*, were most closely related to *Cohnella lupini* LMG 27416^T^ (95.9% and 96.1% similarities), and both showed 94.0% similarity with *Cohnella arctica* NRRL B-59459^T^, respectively. The two strains showed 99.8% 16S rRNA gene sequence similarity between them. The draft genome size of strain NL03-T5^T^ was 7.44 Mbp with a DNA G+C content of 49.2 mol%. The average nucleotide identities (ANI) and the digital DNA–DNA hybridization (dDDH) values between NL03-T5^T^ and NL03-T5-1 were 99.98% and 100%, indicating the two strains were of the same species. Additionally, the ANI and dDDH values between NL03-T5^T^ and *C. lupini* LMG 27416^T^ were 76.1% and 20.4%, respectively. The major cellular fatty acids of strain NL03-T5^T^ included anteiso-C_15:0_ and iso-C_16:0_. The major polar lipids and predominant respiratory quinone were diphosphatidylglycerol (DPG) and menaquinone-7 (MK-7). Based on phylogenetic analysis, phenotypic and chemotaxonomic characterization, genomic DNA G+C content, and ANI and dDDH values, strains NL03-T5^T^ and NL03-T5-1 represent novel species in the genus *Cohnella*, for which the name *Cohnella silvisoli* is proposed. The type strain is NL03-T5^T^ (=GDMCC 1.2294^T^ = JCM 34999^T^). Furthermore, comparative genomics revealed that the genus *Cohnella* had an open pan-genome. The pan-genome of 29 *Cohnella* strains contained 41,356 gene families, and the number of strain-specific genes ranged from 6 to 1649. The results may explain the good adaptability of the *Cohnella* strains to different habitats at the genetic level.

## 1. Introduction

The genus *Cohnella*, which lies within the family Paenibacillaceae of the order Bacillales, was first proposed by Kämpfer et al. with the description of *Cohnella thermotolerans* as the type species [1]. The genus *Cohnella* currently comprised 45 species with valid names listed in the LPSN database (https://www.bacterio.net/-allnamesac.html, accessed on 1 August 2023). Cells were characterized as Gram-stain-positive, spore-forming or non-spore-forming, aerobic or facultatively anaerobic, rods, and motile or non-motile. The major respiratory quinone is menaquinone-7 (MK-7), the predominant polar lipids contain diphosphatidylglycerol (DPG) and phosphatidylethanolamine (PE), and the main fatty acid profiles include iso-C_16:0_, anteiso-C_15:0_ and C_16:0_ [2]. Species of the genus *Cohnella* are widely distributed in various environments such as soil [3,4], rhizosphere or root nodules [5], water [6], green algae [7], animal faeces [8], Siberian permafrost [9] and preserved vegetables [10]. The *Cohnella* species are thought to play an important role in recycling plant biomass within soil, with multiple members of the genus possessing genes for degradation of chitinase, xylan, hemicellulose and cellulose [11,12,13]. During an investigation on the diversity and novelty study of bacterium in soils of the Nanling National Forests, Guangdong Province, PR China, two strains, designated NL03-T5^T^ and NL03-T5-1, were isolated and described as a novel species of the genus *Cohnella* using polyphasic taxonomic studies including phylogenetic analysis, physiological and biochemical characterization, and genomic analysis. Furthermore, comparative genomics of the *Cohnella* strains were used to define the pan-genome, core genome, and unique genes and to assess genetic diversity to understand their adaptability to different habitats.

## 2. Materials and Methods

### 2.1. Strain Isolation and Cultivation

Strains NL03-T5^T^ and NL03-T5-1 were isolated from a soil sample (24°56′16″ N; 113°00′09″ E) collected on 6 October 2020 from the Nanling National Forests, Guangdong Province, China. The two strains were obtained using a standard dilution and plating method. More specifically, a 1 g air-dried soil sample was added to 9 mL of sterile physiological saline. The mixtures were placed in shaker at 160 rpm for 2 h at 30 °C. Then, 10× series (10^−2^, 10^−3^, 10^−4^ and 10^−5^) dilutions were performed separately and spread onto Reasoner’s 2A (R2A; Haibo, Qingdao, China) medium. After cultivation at 30 °C for a week, colonies were picked out and a pure aerobic culture was obtained by repeated subculture of cells from the edge of the colony. Upon purification, strains were stored at −80 °C as a suspension containing 25% glycerol. 

### 2.2. 16S rRNA Gene Sequence and Phylogenetic Analysis

Genomic DNAs of strains NL03-T5^T^ and NL03-T5-1 were extracted from fresh cells grown on R2A agar using a HiPure Bacterial DNA kit (Magen Biotech Co., Ltd., Guangzhou, China) following the manufacturer’s instructions. The 16S rRNA genes of NL03-T5^T^ and NL03-T5-1 were amplified using the extracted genomic DNAs as a template with the universal primers 27F and 1492R [14]. PCR products were sequenced in Majorbio, China. The 16S rRNA genes were aligned in the EzBioCloud (https://eztaxon-e.ezbiocloud.net/) and GenBank (www.ncbi.nlm.nih.gov) databases accessed on 1 August 2023. The 16S rRNA gene sequences were submitted to the National Center for Biotechnology Information (NCBI) database (https://www.ncbi.nlm.nih.gov/genome, accessed on 1 August 2023) under the accession numbers MZ955418 and OQ913505. Phylogenetic trees based on 16S rRNA genes were reconstructed using methods including maximum-likelihood (ML) [15], neighbor-joining (NJ) [16] and minimum-evolution (ME) [17] using MEGA 7.0 software [18]. The topology in each phylogenetic tree was calculated based on 1000 replications and evolutionary distances were calculated using Kimura’s two-parameter model [19]. Based on the phylogenetic analysis, the type strains *Cohnella abietis* HS21^T^, *Cohnella lupini* LMG 27416^T^, and *Cohnella arctica* NRRL B-59459^T^ obtained from the Belgian Co-ordinated Collections of Micro-organisms and the Agricultural Research Service Culture Collection were used as experiment control strains and cultured under optimum conditions.

### 2.3. Morphological, Physiological, and Biochemical Characteristics

The morphological features of strains NL03-T5^T^ and NL03-T5-1 were observed by light microscope (DM6/MC190, Leica, Wetzlar, Germany) and transmission electron microscope (H7650, Hitachi, Tokyo, Japan) with cell growth on R2A agar for 4 d at 30 °C. The growth test was performed on nutrient agar (NA), trypticase soy agar (TSA), MacConkey (Mac) and MD1 agar (6 g of casein peptone, 2 g of soluble starch, 2 g of MgSO_4_·7H_2_O, 0.4 g of CaCl_2_·2H_2_O, 1 L of distilled water, pH 7.2). Growth at different temperatures (10, 15, 20, 25, 30, 35, 37, 40 °C) was tested on R2A agar for 3–4 d. Sodium chloride tolerance was tested at 0, 0.1, 0.2, 0.5, 1.0 and 1.5%, and pH tolerance was performed from 5.0 to 9.5 at intervals of 0.5 pH units according to the method described in [20] on R2A medium for 3–4 d. The Gram-staining reaction was performed by using a bioMérieux Gram stain kit (bioMérieux, Tokyo, Japan) according to the manufacturer’s instructions. Oxidase activity was tested using oxidase test strips [1% (*w*/*v*) tetramethyl-p-phe-nylenediamine, HKM], and catalase activity was determined by bubble production after mixing cells with 3% H_2_O_2_. Gliding motility was checked by observing the edges of colonies formed on 1:6-diluted R2A and using the hanging drop technique as described by Bernardet et al. (2002) [21]. Hydrolyses of casein (1%, *w*/*v*), CM-cellulose (1%, *w*/*v*), Tween 20 (1%, *w*/*v*) and 40 (1%, *w*/*v*) were examined as described by Son et al. [22]. Other physiological properties were examined using API 20NE and API ZYM kits (bioMérieux) according to the manufacturer’s instructions. 

### 2.4. Chemotaxonomic Properties

For cellular fatty acids, polar lipids, and respiratory quinones analysis, strain NL03-T5^T^ and its related species were harvested from R2A agar after being incubated for 4 d at 30 ℃. Fatty acid methyl esters were extracted using the Sherlock Microbial Identification System (MIDI) protocol version 6.1 and analyzed by gas chromatography (model 7890A, Hewlett Packard, Palo Alto, CA, USA) as previously described [23]. The polar lipids were extracted and determined according to the protocol of Tindall et al. [24]. Respiratory quinones were extracted and purified using the method of Minnikin et al. [25] and analyzed using HPLC (UltiMate 3000, 205 Dionex, Thermo Fisher Scientific, Waltham, MA, USA). 

### 2.5. Genome Sequencing, Annotation, and Pan-Genomic Analysis 

The genomic DNAs of strains NL03-T5^T^ and NL03-T5-1 were sequenced on the Illumina HiSeq platform at Shanghai Majorbio Bio-Pharm Technology Co., Ltd. (Shanghai, China). Sequencing reads were assembled into contigs and scaffolds by applying SPAdes version 3.11.1 with default parameters [26], and the sequences were submitted to the NCBI database under the accession numbers JAIOAP000000000 and JASKHM000000000. Genomic annotation was performed using the software Rapid Annotation using Subsystem Technology (RAST) pipeline (http://rast.nmpdr.org/, accessed on 1 August 2023) with default parameters. The genes encoding carbohydrate active enzymes (CAZymes) were identified using the dbCAN2 meta server (http://cys.bios.niu.edu/dbCAN2, accessed on 1 August 2023) with HMMER annotation (E-Value < 1 × 10^−15^, coverage > 0.35), and biosynthetic gene clusters (BGCs) were annotated using antiSMASH bacterial version 6.1.1 (https://antismash.secondarymetabolites.org, accessed on 1 August 2023) with default parameters, respectively. All 27 reference genomes of the genus *Cohnella* were downloaded from the NCBI database. A pan-genome analysis was performed using the bacterial pan-genome analyses tool (BPGA) pipeline [27] with default parameters. Orthologous genes were identified with the USEARCH algorithm using a threshold of 0.5. Core, accessory, and unique genes were functionally annotated using the eggNOG mapper v2 [28]. The data of the pan-genomes were visualized using the ImageGP online database (https://www.bic.ac.cn/ImageGP/, accessed on 1 August 2023). 

### 2.6. OGRI Calculation and Phylogenomic Analysis

Overall genome relatedness indices (OGRI) including the digital DNA–DNA hybridization (dDDH) and average nucleotide identity (ANI) values were calculated using the Genome-to-Genome Distance Calculator (GGDC) (https://ggdc.dsmz.de/, accessed on 1 August 2023) [29] and OrthANIu (www.ezbiocloud.net/tools/ani, accessed on 1 August 2023) [30], respectively. The whole-genome phylogenetic analysis of the genus *Cohnella* was reconstructed based on 92 up-to-date core genes using the software UBCG version 3.0 [31] with a maximum-likelihood algorithm.

## 3. Results

### 3.1. Phylogenetic Analysis Based on 16S rRNA Genes and Genomic Sequences

**Figure 1:**
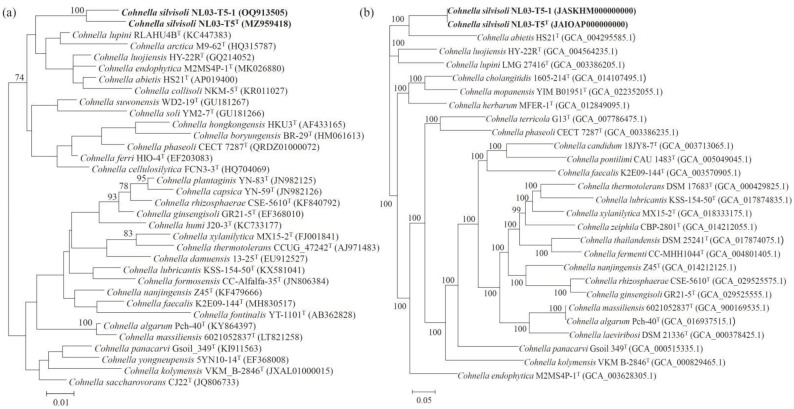
Phylogenetic relationship among species of the genus *Cohnella*. (**a**) Maximum-likelihood phylogenetic tree based on 16S rRNA gene sequences generated by MEGA 7.0 software. Bootstrap values (represented percentages of 1000 replication) > 50% are shown at nodes. Bar, 0.01 substitutions per nucleotide position. (**b**) The UBCG phylogenetic tree based on 92 up-to-date bacterial core genes sequences is constructed using the ML algorithm. Bar, 0.05 substitutions per nucleotide position. GenBank accession numbers are shown in parentheses.

Strains NL03-T5^T^ and NL03-T5-1 showed 99.8% 16S rRNA gene sequence similarity. In the EzBiocloud and NCBI databases, the two strains were closely related to the species of the genus *Cohnella* and showed the highest similarities with *C. lupini* LMG 27416^T^ (95.9% and 96.1%), and they both exhibited 95.3% and 94.0% similarities with *C. abietis* HS21^T^ and *C. arctica* NRRL B-59459^T^, respectively. The 16S rRNA gene phylogenetic trees based on the ML, NJ, and ME methods (Figure 1a and Figures S1 and S2) all showed that strain NL03-T5^T^ and NL03-T5-1 formed an independent cluster with *C. lupini* LMG 27416^T^ and *C. arctica* NRRL B-59459^T^. Furthermore, the phylogenomic tree indicated that strains NL03-T5^T^ and NL03-T5-1 formed an independent cluster with *Cohnella abietis* HS21^T^ (Figure 1b). Therefore, we further selected *C. lupini* LMG 27416^T^, *C. abietis* HS21^T^, and *C. arctica* NRRL B-59459^T^ as reference type strains for taxonomic studies.

### 3.2. Physiological Characterization

**Figure 2:**
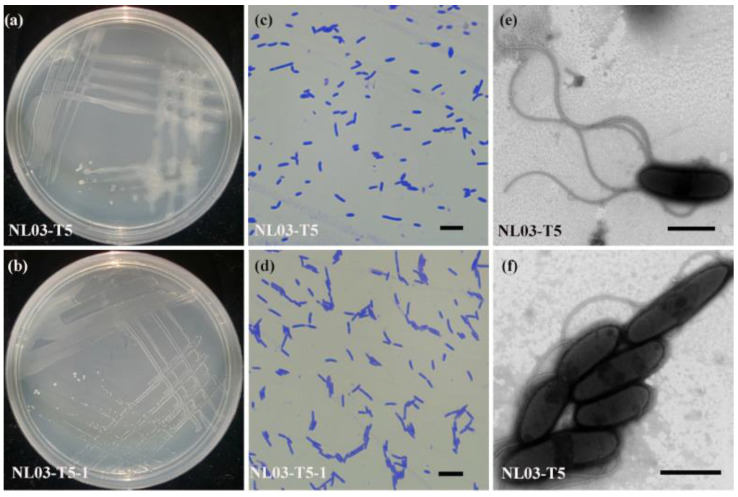
Morphologies of NL03-T5^T^ and NL03-T5-1. (**a**,**b**) Photographs of strains NL03-T5^T^ and NL03-T5-1 grown on R2A for 4 d at 30 °C. (**c**,**d**) Light micrographs of NL03-T5^T^ and NL03-T5-1, cells were stained by crystal violet. Scale: 5 μm. (**e**,**f**) Transmission electronic micrographs of NL03-T5^T^. Scale: 500 nm.

The cells of strains NL03-T5^T^ and NL03-T5-1 were Gram-stain-positive, aerobic, lophotrichous flagellation, rod-shaped, and 1.2–2.0 μm long and 0.4–0.6 μm in diameter after incubation on R2A agar for 4 d at 30 °C (Figure 2c–f). Colonies were white-cream colored and strain NL03-T5^T^ could secrete extracellular mucus whereas NL03-T5-1 could not (Figure 2a,b). The two strains could grow on R2A, but not on NA, TSA, Mac and MD1 agar. Physiological analyses indicated that strains NL03-T5^T^ and NL03-T5-1 were able to grow at 10–37 °C, pH 5.0–8.5 and cells could tolerate 0.5% (*w*/*v*) NaCl (Table 1). Strains NL03-T5^T^ and NL03-T5-1 were negative for oxidase activity whereas their closely related species *C. abietis* HS21^T^, *C. lupini* LMG 27416^T^, and *C. arctica* NRRL B-59459^T^ were positive. In addition, strain NL03-T5^T^ was positive for hydrolysis of Tween 20 and utilization of *a*-mannosidase whereas its related species *C. lupini* LMG 27416^T^ and *C. arctica* NRRL B-59459^T^ were negative. Additional differences between strain NL03-T5^T^ and its closely related species are shown in Table 1. 

### 3.3. Chemotaxonomic Analysis

The cellular fatty acid compositions of strain NL03-T5^T^ and its reference species *C. abietis* HS21^T^, *C. lupini* LMG 27416^T^, and *C. arctica* NRRL B-59459^T^ are given in Table 2. The whole-cell fatty acids of strain NL03-T5^T^ contained a large amount of anteiso-C_15:0_ (50.7%) and iso-C_16:0_ (21.2%), and small amounts of C_16:0_ (7.2%), iso-C_15:0_ (5.5%), anteiso-C_17:0_ (4.0%), iso-C_14:0_ (3.7%), iso-C_14:0_ (1.9%), anteiso-C_13:0_ (1.6%) and C_14:0_ (1.3%). The prominent fatty acids were similar to other species of the genus *Cohnella*, indicating that strain NL03-T5^T^ is a member of the genus *Cohnella*. However, strain NL03-T5^T^ contained a higher amount of iso-C_16:0_ than its closely related species *C. abietis* HS21^T^, and it contained a lower amount of C_16:0_ and a higher amount of iso-C_15:0_ and iso-C_16:0_ than *C. lupini* LMG 27416^T^ and *C. arctica* NRRL B-59459^T^. Moreover, strain NL03-T5^T^ did not contain alcohol-C_16:1_ *ω7c* (6.5%) or C_16:1_ *ω11c* (7.0%) whereas *C. lupini* LMG 27416^T^ did. These significant differences in the fatty acids clearly distinguished strain NL03-T5^T^ from other related species. The major polar lipids of strain NL03-T5^T^ were DPG and PE, which were in common with its closely related species. Otherwise, four unidentified amino phospholipids (APLs) were also detected in strain NL03-T5^T^ (Figure S3). The predominant menaquinone of strain NL03-T5^T^ was MK-7, which was found in all members of the genus *Cohnella* [2]. 

### 3.4. Genomic Characteristics and OGRI Values

The draft genome size of strain NL03-T5^T^ was 7.44 Mbp with 43 contigs and an N50 value of 310,309 bp. In addition, the draft genome size of strain NL03-T5-1 was 7.44 Mbp with 41 contigs and an N50 value of 310,325. The two genomics DNA G+C contents were both 49.2 mol%, which were lower than the G+C contents of *C. lupini* LMG 27416^T^ and *C. arctica* NRRL B-59459^T^ (50.7 and 50.3 mol%), and higher than *C. abietis* HS21^T^ (44.8%) (Table 1). The distribution of genes into functional categories of strain NL03-T5^T^ using RAST revealed that the highest percentages of genes were assigned to carbohydrates (18.5%), amino acids and derivatives (17.3%), protein metabolism (9.3%) and cofactors, vitamins, prosthetic groups, and pigments (8.9%) (Table S1). In addition, strains NL03-T5^T^ and NL03-T5-1 showed 20 gene differences in the functional category of amino acids and derivatives, which probably cause phenotypic differences between the two strains (Table S1). The antiSMASH tool identified four complete BGCs and four BGCs on the contig edge that might be fragments of BGCs. The four complete BGCs include a resorcinol, a terpene, a RiPP-like and a phosphonate. The dbCAN2 analysis of the NL03-T5^T^ genome predicted 513 CAZymes which were distributed across 114 different CAZymes families, with glycoside hydrolases (GHs) and carbohydrate-binding modules (CBMs) constituting the most abundant families (Figure S4). Additionally, the ANI and dDDH values of strain NL03-T5^T^ and NL03-T5-1 were 99.98% and 100%, indicating the two strains were of the same species. The ANI values among NL03-T5^T^ and its closely related species *C. abietis* HS21^T^ and *C. lupini* LMG 27416^T^ were 75.7% and 76.0%, respectively. The dDDH values among them both were 20.4%. These values were lower than the threshold values of 95–96% and 70% for species discrimination [33], indicating that strains NL03-T5^T^ and NL03-T5-1 represent a novel species. 

### 3.5. Pan-Genome Analysis of the Genus Cohnella

**Figure 3:**
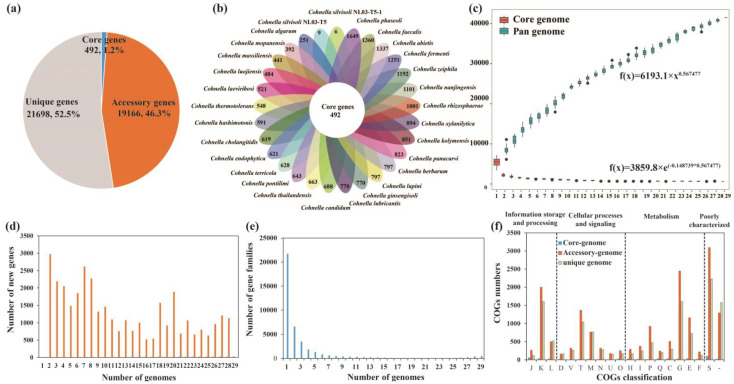
The pan-genome analysis of the genus *Cohnella*. (**a**) The numbers and proportions of core genes, accessory genes, and unique genes. (**b**) Flower plot painting the core genome and unique-specific strain of the *Cohnella* strains. (**c**) Boxplots of the pan-genome (blue) and core genome (red) of the 29 analyzed genomes. (**d**) Number of new genes represented within the numbers of *Cohnella* genomes. (**e**) Number of gene families represented within the numbers of *Cohnella* genomes. (**f**) The proportions of COGs functional categories of core genes, accessory genes, and unique genes. J, translation, ribosomal structure and biogenesis; K, transcription; L, replication, recombination, and repair; D, cell cycle control, cell division, and chromosome partitioning; V, defense mechanisms; T, signal transduction mechanisms; M, cell wall/membrane/envelope biogenesis; N, cell motility; U, intracellular trafficking, secretion, and vesicular transport; O, posttranslational modification, protein turnover, and chaperones; H, coenzyme transport and metabolism; I, lipid transport and metabolism; P, inorganic ion transport and metabolism; Q, secondary metabolite biosynthesis, transport, and catabolism; C, energy production and conversion; G, carbohydrate transport and metabolism; E, amino acid transport and metabolism; F, nucleotide transport and metabolism; S, function unknown; -, general function prediction only.

The pan-genome of the 29 *Cohnella* strains comprised 41,356 gene families. The core genes were present in all 29 genomes, accessory genes were present in 2–28 genomes, and unique genes were present only in one genome. The numbers of core genes, accessory genes, and unique genes were 492 (1.2%), 19,166 (46.3%), and 21,698 (52.5%), respectively (Figure 3a). The numbers of strain-specific genes ranged from 6 to 1649 (Figure 3b), suggesting there is an obvious difference among the genomes of the 29 *Cohnella* strains. The size of the pan-genome increased with the increasing number of genomes. Correspondingly, the core-genome size decreased with the addition of genomes (Figure 3c). The curves of pan-genome and core-genome sizes indicated an open pan-genome of the genus *Cohnella*, which was supported by the parameter b value (0.567477, between zero and one) in the power-law regression function. New gene distribution and gene family distribution of the 29 *Cohnella* strains are shown in Figure 3d and Figure 3e, respectively. Functional characterization from core, accessory, and unique genes was conducted using the COGs annotation. As shown in Figure 3f, many core, accessory, and unique genes were assigned to the category “S”, indicating their functions await to be studied further. Except for poorly characterized categories, the largest proportion of core genes belonged to the categories “translation, ribosomal structure and biogenesis (J)”, followed by “amino acid transport and metabolism (E)”. In contrast, the highest percentage of accessory genes and unique genes was related to carbohydrate transport and metabolism (G), followed by transcription (K). The results above may explain the good adaptability of the *Cohnella* strains to different habitats through gene gains or losses during frequent evolutionary changes at the genetic level. 

## 4. Discussion

In this study, we isolated two bacterial strains, designated NL03-T5^T^ and NL03-T5-1, from a soil sample collected from the Nanling National Forests, PR China. The two strains showed 99.8% 16S rRNA gene sequence similarity. The ANI and dDDH values between them are 99.98% and 100%. These results indicate the two strains are of the same species. However, strain NL03-T5^T^ could secrete extracellular mucus whereas NL03-T5-1 could not (Figure 2a,b). Furthermore, the distribution of genes into functional categories using RAST revealed 20 gene differences in the functional category of amino acids and derivatives between them (Table S1). These different genes probably cause phenotypic differences. Based on phylogenetic analysis, genomic DNA G+C content, ANI and dDDH values, physiological characterization and chemotaxonomic analysis, strain NL03-T5^T^ was identified as a novel species in the genus *Cohnella*, for which the name *Cohnella silvisoli* sp. nov. is proposed. 

The genus *Cohnella* strains are widely distributed in different environments. This to a certain extent can be explained by the nature of *Cohnella* which allows it to acclimatize itself to many environments. Comparative genomics indicated that the genus *Cohnella* had an open pan-genome and exhibited broad genetic diversity. In general, an open pan-genome is predominant in bacteria that are susceptible to horizontal gene transfer (HGT) [34]. The pan-genome of 29 *Cohnella* strains contained 41,356 gene families, and the numbers of core genes, accessory genes, and unique genes were 492, 19,166, and 21,698, respectively (Figure 3a,c). Tettelin et al. (2008) has illustrated that the core genome is essential for the basic lifestyle of bacteria, whereas the accessory genome and unique genes provide some characteristics such species diversity and environmental adaptability [35]. Therefore, we preliminarily inferred that the genomic differences in the *Cohnella* strains might be associated with their colonized environments.

## 5. Description of *Cohnella silvisoli* sp. nov.

*Cohnella silvisoli* (sil.vi.so′li. L. fem. n. silva forest; L. neut. n. solum soil; N.L. gen. n. silvisoli of forest soil, the source of isolation of the type strain).

Cells are aerobic, Gram-stain-positive, lophotrichous flagellation, rod-shaped, 1.2–2.0 μm long and 0.4–0.6 μm in diameter after incubation on R2A agar for 4 d. Colonies on R2A agar are white-cream, circular, convex with extracellular secretions or not. Growth occurs at 15–37 ℃ and at a pH range from 5.0 to 8.5 (optimum, pH 7.0). Cells can tolerate 0.5% (*w*/*v*) NaCl. Oxidase and catalase are -negative. It can hydrolyze Tween 20, but not Tween 40, casein, or CM-cellulose. Additionally, it can hydrolyze gelatin or not. The major fatty acids (>10%) include anteiso-C_15:0_ (50.7%) and iso-C_16:0_ (21.2%). The predominant polar lipid is DPG. The main respiratory quinone is MK-7. The API ZYM test result was positive for alkaline phosphatase, esterase, lipase, leucine arylamidase, acid phosphatase, naphthol-AS-BI-phosphohydrolase, *α*-galactosidase, *β*-galactosidase, *α*-glucosidase, *β*-glucosidase, *N*-acetyl-*β*-glucosaminidase and *α*-mannosidase; but negative for valine arylamidase, cystine arylamidase, trypsin, *α*-chymotrypsin and *α*-fucosidase. The API 20NE test results were positive for gelatin hydrolysis, 4-nitrophenyl-*β*-d-galactopyranosidase and *β*-glucosidase or not; but negative for nitrate reduction, indole production, glucose fermentation, arginine dihydrolase and urease. It can assimilate glucose, *L*-arabinose, mannose, mannitol, *N*-acetyl-D-glucosamine, maltose, gluconate, adipate, malic acid, citric acid, phenylacetic acid and capric acid or not.

The type strain is NL03-T5^T^ (=GDMCC 1.2294^T^ = JCM 34999^T^), which was isolated from a soil sample collected from the Nanling National Forests, Guangdong Province, PR China. The genomic DNA G+C content of the type strain is 49.2 mol%.

## Figures and Tables

**Table 1:** Differential phenotypic characteristics of strains NL03-T5^T^, NL03-T5-1 and their closely related species of the genus *Cohnella*.

Characteristics	NL03-T5^T^	NL03-T5-1	*C. abietis* HS21^T^ [32]	*C. lupini* LMG 27416^T^	*C. arctica* NRRL B-59459^T^
Isolation source	soil	soil	soil	root nodules of *Lupinus albus*	soil
Colony colour	white-cream	white-cream	white	orange	white-cream
Oxidase	−	−	+	+	+
Catalase	−	−	−	+	+
Growth at 37 °C	w	+	−	−	−
Temperature range	10–37	10–37	4–30	10–35	10–35
pH range	5.0–8.5	5.0–8.5	6–8	5.5–8.5	5.0–9.0
**Growth on media:**					
Reasoner’s 2A	+	+	+	−	+
NA	−	−	+	+	−
**Hydrolysis of:**					
Gelatin	+	+	−	+	+
Tweens 20	+	+	ND	−	−
**Assimilation of:**					
Mannitol	+	+	+	−	+
*N*-Acetyl-D-glucosamine	+	+	+	−	+
Gluconate	+	+	−	−	+
Capric acid	−	+	−	−	+
**Enzyme activities:**					
Esterase (C8)	+	+	ND	−	+
*β*-Glucosidase	+	−	ND	+	+
*α*-Mannosidase	+	+	ND	−	−
GC content (mol%)	49.2	49.2	44.8	50.7	50.3

All data are from this study unless indicated otherwise. +, positive; −, negative; w, weakly positive reaction; ND, no data available.

**Table 2:** Cellular fatty acids of strain NL03-T5^T^ and its closely related species of the genus *Cohnella*. Strains: 1, NL03-T5^T^; 2, *C. abietis* HS21^T^ [32]; 3, *C. lupini* LMG 27416^T^; 4, *C. arctica* NRRL B-59459^T^. Data of strain 1, 3 and 4 were from this study. All strains were incubated on R2A agar for 4 d at 30 °C. Values are percentages of the total fatty acids. Fatty acids that make up <1.0% of the total are not shown. -, not detected or <1.0%.

Fatty Acid	1	2	3	4
anteiso-C_13:0_	1.6	4.3	-	2.1
iso-C_14:0_	3.7	2.7	1.1	2.6
C_14:0_	1.3	1.6	2.3	3.3
iso-C_15:0_	5.5	6.0	2.1	2.1
anteiso-C_15:0_	50.7	50.9	42.2	44.7
C_16:0_	7.2	9.9	22.0	14.5
alcohol-C_16:1_ ω7c	-	-	6.5	-
C_16:1_ ω*11c*	-	-	7.0	-
iso-C_16:0_	21.2	13.7	6.9	18.0
iso-C_17:0_	1.9	2.4	1.2	-
anteiso-C_17:0_	4.0	3.2	5.2	4.1
C_17:0_	-	-	-	2.1
C_18:0_	-	-	-	1.4
Summed feature 8	-	-	-	1.4

Summed features are fatty acids that cannot be resolved reliably from another fatty acid using the chromatographic conditions chosen. The MIDI system groups these fatty acids together as one feature with a single percentage of the total. Summed feature 8, C_18:1_ *ω*7c and/or C_18:1_ *ω6c.*

## Data Availability

Not applicable.

## Supplementary Materials

The following supporting information can be downloaded at: https://www.mdpi.com/article/10.3390/microorganisms11112726/s1, Figure S1: The neighbor-joining tree based on 16S rRNA gene sequences constructed revealing the phylogenetic position of strain NL03-T5^T^, NL03-T5-1 and its closely related species of the genus *Cohnella*. Only the bootstrap values (represented percentages of 1000 replication) ˃50% are shown. GeneBank accession numbers are shown in parentheses. Bar, 0.005 substitutions per nucleotide position; Figure S2: The minimum evolution tree based on 16S rRNA gene sequences constructed indicating the relationship of strain NL03-T5^T^, NL03-T5-1 and its closely related species of the genus *Cohnella*. Bootstrap values (represented percentages of 1000 replication) ˃50% are shown at nodes. GenBank accession numbers are shown in parentheses. Bar, 0.005 substitutions per nucleotide position; Figure S3: Two-dimensional thin-layer chromatograms of the polar lipids of strain NL03-T5^T^ and its closely related species, *C. lupini* LMG 27416^T^ and *C. arctica* NRRL B-59459^T^, by spraying with 5% ethanolic molybdatophosphoric acid and heating them at 140 °C for 15 min. Abbreviations: DPG, diphosphatidylglycerol; PE, phosphatidylethanolamine; PG, phosphatidylglycerol; APL, unidentified aminophospholipid; Figure S4: Total CAZymes in the various families, predicted in NL03-T5^T^ genome; Table S1: Genome characteristics of *Cohnella silvisoli* NL03-T5^T^ and NL03-T5-1.

## Abbreviations

GDMCC	Guangdong Microbial Culture Collection Center
JCM	Japan Collection of Microorganism
LMG	Belgian Co-ordinated Collections of Micro-organisms
NRRL	Agricultural Research Service Culture Collection
ANI	Average Nucleotide Identity
dDDH	the Digital DNA-DNA Hybridization
UBCG	Up-to-date Bacterial Core Gene set
DPG	diphosphatidylglycerol
PE	phosphatidylethanolamine
PG	phosphatidylglycerol
APL	unidentified aminophospholipid
