# Supplementary material for: Pan-Genome Analyses of the Genus Cohnella and Proposal of the Novel Species Cohnella silvisoli sp. nov., Isolated from Forest Soil
Source: Microorganisms. 2023 Nov 8;11(11):2726. doi: 10.3390/microorganisms11112726 (PMC10672984; doi:10.3390/microorganisms11112726)
Supplement: Supplementary file 1 [file microorganisms-11-02726-s001.zip › microorganisms-2607021-supplementary.pdf]

Supplementary data

Pan-Genome Analyses of the Genus *Cohnella* and Proposal of the Novel Species *Cohnella silvisoli* sp. nov., Isolated from Forest Soil

Chunling Wang [1], Lutian Mao [1], Gegen Bao [2] and Honghui Zhu [3,*]

[1] College of Life Science, Huizhou University, Huizhou 516007, China; wangcl@hzu.edu.cn (C.W.); mlt@hzu.edu.cn (L.M.)
[2] Guangzhou Key Laboratory for Research and Development of Crop Germplasm Resources,
Zhongkai University of Agriculture and Engineering, Guangzhou 510225, China; baogegen@126.com
[3] Key Laboratory of Agricultural Microbiomics and Precision Application (MARA),
Provincial Key Laboratory of Microbial Culture Collection and Application,
Key Laboratory of Agricultural Microbiome (MARA),
State Key Laboratory of Applied Microbiology Southern China, Institute of Microbiology, Guangdong Academy of Sciences, Guangzhou 510642, China
* Correspondence: zhuhh_gdim@163.com; Tel.: +86-020-87685669

Subject Category: New Taxa (*Firmicutes*)

Article Type: Taxonomic Descriptions

Abbreviations: GDMCC, Guangdong Microbial Culture Collection Center; JCM, Japan Collection of Microorganism; LMG, Belgian Co-ordinated Collections of Micro-organisms; NRRL, Agricultural Research Service Culture Collection; ANI, Average Nucleotide Identity; dDDH, the Digital DNA-DNA Hybridization; UBCG, Up-to-date Bacterial Core Gene set; DPG, diphosphatidylglycerol; PE, phosphatidylethanolamine; PG, phosphatidylglycerol; APL, unidentified aminophospholipid.

Figure S1. The neighbor-joining tree based on 16S rRNA gene sequences constructed revealing the phylogenetic position of strain NL03-T5^T, NL03-T5-1 and its closely related species of the genus *Cohnella*. Only the bootstrap values (represented percentages of 1000 replication) >50% are shown. GeneBank accession numbers are shown in parentheses. Bar, 0.005 substitutions per nucleotide position.

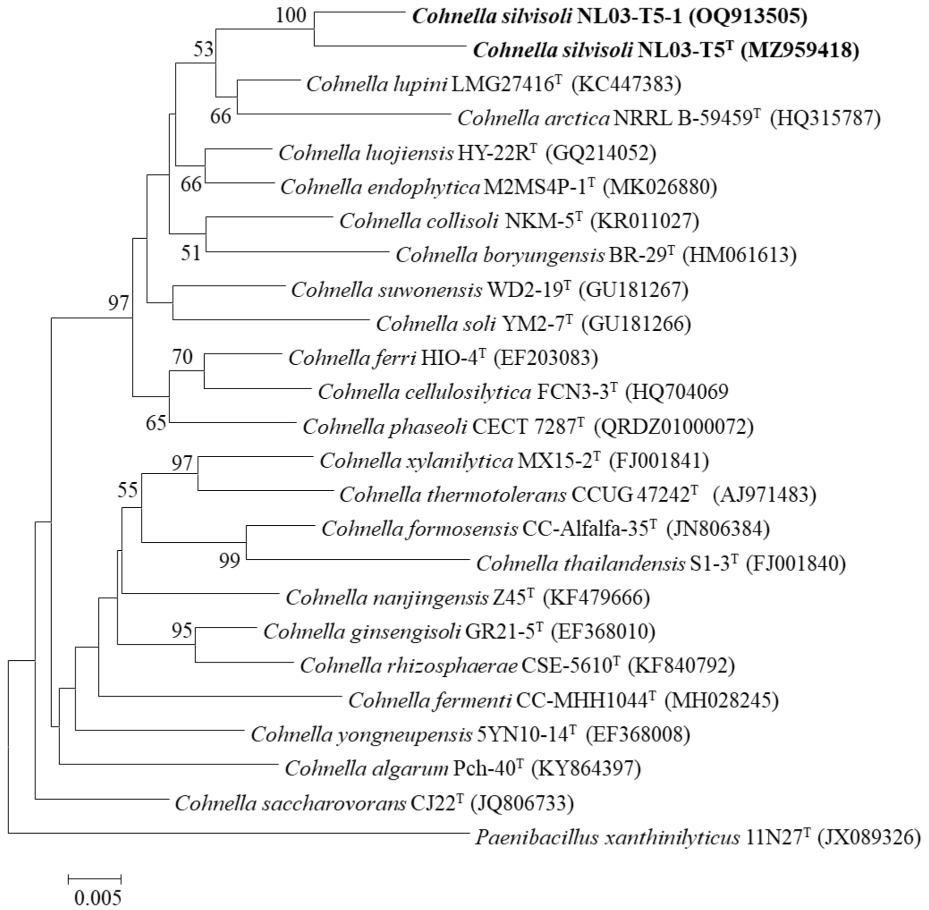

Figure S2. The minimum evolution tree based on 16S rRNA gene sequences constructed indicating the relationship of strain NL03-T5^T, NL03-T5-1 and its closely related species of the genus *Cohnella*. Bootstrap values (represented percentages of 1000 replication) >50% are shown at nodes. GenBank accession numbers are shown in parentheses. Bar, 0.005 substitutions per nucleotide position.

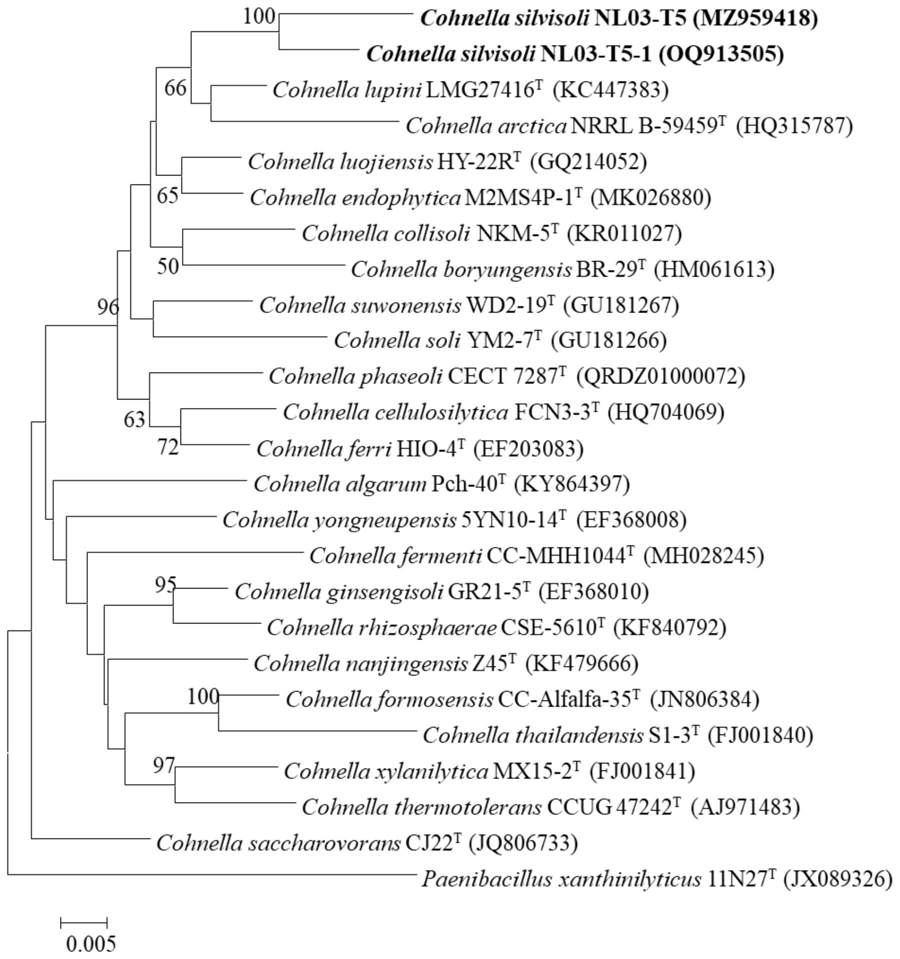

Figure S3. Two-dimensional thin-layer chromatograms of the polar lipids of strain NL03-T5^{T} and its closely related species, *C. lupini* LMG 27416^{T} and *C. arctica* NRRL B-59459^{T}, by spraying with 5% ethanolic molybdatophosphoric acid and heating them at 140°C for 15 min. Abbreviations: DPG, diphosphatidylglycerol; PE, phosphatidylethanolamine; PG, phosphatidylglycerol; APL, unidentified aminophospholipid.

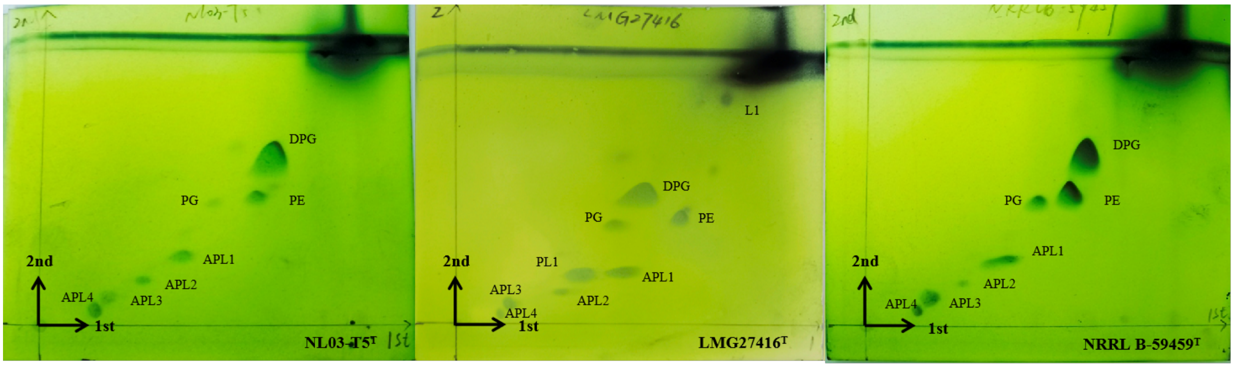

Figure S4. Total CAZymes in the various families, predicted in NL03-T5^{T} genome.

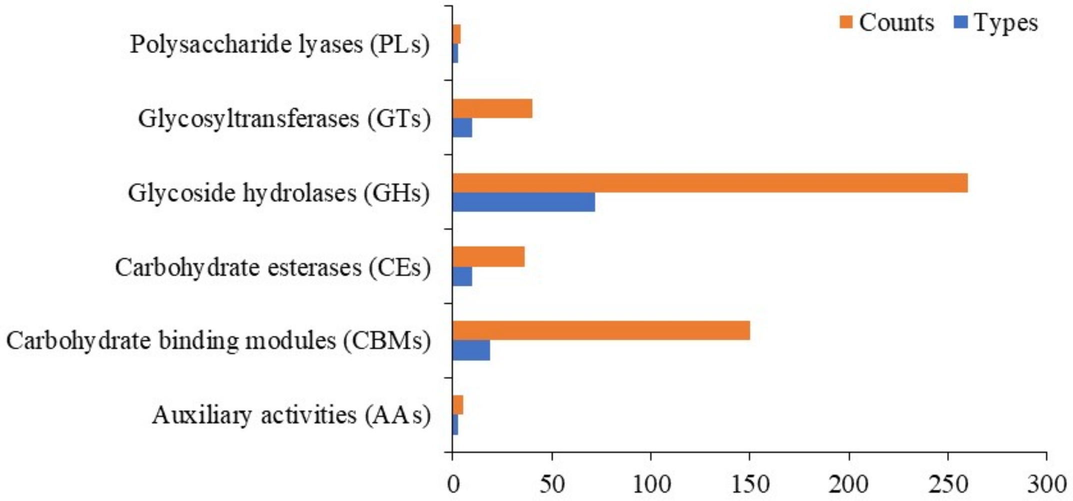

Table S1. Genome characteristics of *Cohnella silvisoli* NL03-T5^T and NL03-T5-1.

Characteristics	NL03-T5^T	NL03-T5-1
Accession No.	JAIOAP000000000	JASKHM000000000
Size (Mbp)	7.44	7.44
No. of contigs	43	41
%GC content	49.2	49.2
No. Of coding sequences	6853	6860
L50 (bp)	8	9
N50 (bp)	310309	310325
Number of RNAs	67	66
Completeness (%)	91.2	92.5
Contamination (%)	15.4	14.1
**Number of genes related to (RAST):**		
Cofactors, vitamins, prosthetic groups, pigments	138	138
Cell wall and capsule	55	55
Virulence, disease, and defense	56	55
Potassium metabolism	7	7
Miscellaneous	11	11
Phages, prophages, transposable elements, plasmids	9	9
Membrane transport	23	24
Iron acquisition and metabolism	19	19
RNA metabolism	55	55
Nucleosides and nucleotides	96	96
Protein metabolism	145	145
Cell division and cell cycle	6	6
Motility and chemotaxis	19	19
Regulation and cell signaling	17	17
Secondary metabolism	6	6
DNA metabolism	79	79
Fatty acids, lipids, and isoprenoids	40	41
Nitrogen metabolism	8	8
Dormancy and sporulation	25	25
Respiration	60	60
Stress response	39	39
Metabolism of aromatic compounds	15	15
Amino acids and derivatives	268	248
Sulfur metabolism	17	17
Phosphorus metabolism	52	52
Carbohydrates	287	287
**Region (antiSMASH):**	**Type**	**Type**
1.1	resorcinol	resorcinol
2.1	RiPP-like	RiPP-like
2.2	NRPS	NRPS

5.1	terpene	terpene
17.1	RiPP-like	RiPP-like
24.1	terpene	terpene
25.1	NRPS	NRPS
29.1	phosphonate	phosphonate
37.1	NRPS	NRPS
